# Decoding Speech Perception by Native and Non-Native Speakers Using Single-Trial Electrophysiological Data

**DOI:** 10.1371/journal.pone.0068261

**Published:** 2013-07-11

**Authors:** Alex Brandmeyer, Jason D. R. Farquhar, James M. McQueen, Peter W. M. Desain

**Affiliations:** 1 Donders Institute for Brain, Cognition and Behaviour, Radboud University Nijmegen, Nijmegen, The Netherlands; 2 Behavioural Science Institute, Radboud University Nijmegen, Nijmegen, The Netherlands; 3 Max Planck Institute for Psycholinguistics, Nijmegen, The Netherlands; University of Maryland, College Park, United States of America

## Abstract

Brain-computer interfaces (BCIs) are systems that use real-time analysis of neuroimaging data to determine the mental state of their user for purposes such as providing neurofeedback. Here, we investigate the feasibility of a BCI based on speech perception. Multivariate pattern classification methods were applied to single-trial EEG data collected during speech perception by native and non-native speakers. Two principal questions were asked: 1) Can differences in the perceived categories of pairs of phonemes be decoded at the single-trial level? 2) Can these same categorical differences be decoded across participants, within or between native-language groups? Results indicated that classification performance progressively increased with respect to the categorical status (within, boundary or across) of the stimulus contrast, and was also influenced by the native language of individual participants. Classifier performance showed strong relationships with traditional event-related potential measures and behavioral responses. The results of the cross-participant analysis indicated an overall increase in average classifier performance when trained on data from all participants (native and non-native). A second cross-participant classifier trained only on data from native speakers led to an overall improvement in performance for native speakers, but a reduction in performance for non-native speakers. We also found that the native language of a given participant could be decoded on the basis of EEG data with accuracy above 80%. These results indicate that electrophysiological responses underlying speech perception can be decoded at the single-trial level, and that decoding performance systematically reflects graded changes in the responses related to the phonological status of the stimuli. This approach could be used in extensions of the BCI paradigm to support perceptual learning during second language acquisition.

## Introduction

Learning foreign languages is difficult, in part because they often make use of sounds which are unfamiliar. Moreover, foreign speech sounds can be difficult to discriminate from one another, depending on the types of phonemes used in one’s native language. Studies of human language perception have made use of EEG measurements to reveal differences in the processing of speech sounds by the brains of native and non-native listeners. The results of these studies are typically based on the analysis of event-related potentials collected over hundreds of trials and using many individual participants. This is done because the signals of interest are much smaller in amplitude than the ongoing brain activity measured during single-trials [1]. By averaging EEG data collected during repeated time-locked presentations of speech sounds, brain activity unrelated to the stimulus presentation is eventually cancelled out, leaving only the brain’s responses to the speech sound. But what if it were possible to detect these signals in single-trial EEG data? Research using multivariate pattern classification methods and brain-computer interface (BCI) paradigms has shown that this is feasible for signals such as the P3 response [2], [3]. In turn, users are able to control different types of systems (e.g. communication devices and computers) using mental activity alone by, for instance, attending to items in a flashing menu. If it was also possible to detect the brain responses underlying speech perception, it could allow for the development of BCIs that support the learning of foreign languages through the monitoring of ongoing perception, or by providing feedback to users on their brain’s responses.

To this end, a study was conducted using a multivariate analysis of EEG data collected during passive auditory perception of English language phonemes by native and non-native speakers of English. It investigated whether such methods are sensitive to the different electrophysiological response patterns elicited when native and non-native listeners are presented with pairs of stimuli from a continuum of phonemes representing either within- or across-category contrasts. Additionally, the study used the same methods in conjunction with two cross-participant data sets to address questions regarding the consistency of the functional brain organization underlying speech perception across individuals both within and between language groups.

### The Mismatch Negativity Component and Research on Speech Perception

Previous research using auditory event-related potentials (ERPs) has revealed consistent differences between native and non-native speakers in the brain responses underlying the perception of phonetic contrasts [4]–[6]. These findings are often based on analysis of the mismatch negativity (MMN) component of the auditory ERP, which is typically seen at fronto-central scalp locations following the presentation of a low-probability ‘deviant’ stimulus. As the MMN is typically elicited using a passive listening paradigm, it is thought to provide a pre-attentive index of perceptual discrimination abilities [7], [8]. In addition, the amplitude and latency of the MMN have been shown to be modulated by the stimulus contrast employed. Large differences between standard and deviant stimuli will lead to increases in MMN amplitude as well as a decrease in its latency, while smaller differences will reduce the amplitude and increase the latency [9].

The MMN has been observed in response to both changes in acoustic features of phonemes typical of within-category variation [10]–[12] as well as when presenting stimulus contrasts representing two distinct phonemic categories [4]–[6], [10], [13], [14]. A comparison of the MMN responses evoked by stimuli from a phonetic continuum containing both within- and across-category deviants showed that across-category responses were significantly larger than within-category responses [10]. When non-native listeners are presented with a meaningful phonetic contrast in an unfamiliar language, the measured ERPs typically show a reduced [4] or absent MMN response [5], [6], [13] relative to native speakers. Thus it would seem that MMN responses observed in response to phonemes show a graded effect, with respect to both the categorical status of the phonetic contrast as well as to the linguistic background of individual listeners.

While MMN responses to artificial tone stimuli are consistently reported in the N1 interval [7], studies using phonetic stimuli have reported MMN in both the N1 [4], [12] and N2 [6], [10] intervals. It has been suggested that stimulus contrasts representing distinct phonetic categories give rise to changes in the N2-P3 complex of the auditory ERP, while effects in the N1 interval reflect the processing of acoustic differences in the stimuli [15]. Other findings have also suggested a distinction between early and late MMN responses to speech [16] and speech-like [17] stimuli. Additionally, the same auditory oddball paradigms used to elicit MMN responses have also been shown to modulate mid-latency components prior to the N1 [18], and to elicit a negative component following the P3a response known as the reorienting negativity (RON) [19], [20]. As such, depending on stimulus and sequence parameters, ERPs collected on deviant trials during MMN measurement paradigms can be expected to show an enhancement of negative components in one or more time intervals relative to standard trials. The question we asked here was whether these (or other) components could be detected reliably at the single-trial level.

### Multivariate Analysis Methods and Auditory Perception

While the neurophysiology of speech perception has been examined extensively using traditional ERP methodologies, there has recently been an increasing interest in the use of multivariate pattern classification methods to address questions regarding the functional organization of cognitive processes using data collected at the single-trial level [21]–[25], and to develop BCIs based on real-time measurementsof brain activity. Whereas the traditional univariate methods used to analyze neurophysiological signals such as the BOLD response or ERP measurements focus on amplitude differences at individual data points (i.e. sensors, time points, voxels), multivariate methods are sensitive to differences in the distribution of responses across high-dimensional feature spaces. Moreover, when used with data collected at the single-trial level, additional information contained within the single-trial responses is available which might otherwise be lost when averaging across trials.

Several BCI studies have used multivariate methods to detect different classes of auditory ERPs elicited by target and non-target stimuli in an active task. Such tasks are know to elicit a P3 response [8], [26], and have also been used with stimuli in the visual [2], [3] and tactile [27] modalities. Halder and colleagues reported on a system capable of making binary choices using auditory targets which differed in either loudness, pitch or direction [28]. Systems capable of distinguishing a larger number of classes using either spatial [29], [30] or a combination of spatial and frequency [31] cues have also been reported. Additional work has shown that the use of speech stimuli can enhance classifier performance relative to artificial stimuli [32]. While the principal focus in these studies has been the elicitation of a P3 response for use as a control signal in determining whether a target stimulus has been presented, some of the studies just mentioned have also reported on the contribution of negative ERP components in the 100–300 ms post-stimulus onset time interval to overall BCI performance [29], [31].

Multivariate approaches have also been used in several studies to investigate auditory perception of speech and music at the single-trial level. In the music domain, it has been shown that decoding perceived music from EEG data at the single trial level is possible, and that decoding using cross-participant data sets leads to similar overall performance as compared to within-participant analyses [33]. Additional work using EEG data has shown that the decoding of accented vs. unaccented beats in an isochronous sequence is possible, during both active perception as well as during a subjective-accenting task, and that decoding performance generalizes across these conditions [34]. With regard to speech perception, it has been shown that the brain activity underlying the perception of different vowels and different speaking voices can be decoded from single-trial fMRI data [24]. A recent study by Herrmann and colleagues demonstrated that both unexpected changes in low-level acoustic features as well as syntactic-rule violations can be also decoded using MEG data, with cross-participant analyses showing a high-degree of consistency in both the spatial distribution of features as well as in overall performance relative to individual analyses [35].

### The Present Study

Here, we aim to extend these findings by examining whether the perception of phonetic contrasts representing within- or across-category contrasts can be decoded using single-trial EEG data. This is accomplished using a dataset from a recently published study on within- and between-group differences in the perception of a phonetic continuum by native (English) and non-native (native-Dutch) speakers [36]. This makes it possible to interpret the results of the present classification analyses with respect to outcomes of traditional ERP analyses as well as individual behavioral measurements. In addition to the within-participant analyses, we also present the results of both multi-trial and cross-participant decoding analyses, and, on the basis of these results, discuss the potential for novel extensions of the BCI paradigm to the domain of second language learning.

## Materials and Methods

### Ethics Statement

All participants provided written informed consent prior to their participation in the experiment. The experiment was performed in accordance with the guidelines of and was approved by the ethics committee of the Faculty of Social Sciences, Radboud University Nijmegen.

### Participants and Stimuli

The present study was a reanalysis of the data collected in [36] during passive speech perception of English language phonemes by native and non-native speakers of English. The non-native speakers who participated in the experiment were all native speakers of Dutch, and were also proficient speakers of English, having undergone at least 6 years of English language education. We will refer to the native speakers as ‘native-English’ and the non-native speakers as ‘native-Dutch’. Data for the same eleven participants in each of the two language groups as in the original study were used. A summary of the experimental design can be found in Table 1.

**Table 1:** Details of experimental paradigm.

Participants	11 Native-English speakers, 11 Native-Dutch speakers
Stimuli	English language CV (/pa/−/ba/) syllables: 85 ms VOT (standard)
	63 ms, 41 ms and 19 ms VOT (deviants)
Stimulus Intensity	approx. 70 dB
Stimulus Duration	approx. 450 ms
ISI	1200 ms
Deviant Likelihood	15%
Trial Counts	90–135 per deviant condition, per participant
EEG System	64 Channel BioSemi Active2+ left & right mastoids,
	Horizontal & Vertical EOG
ERP analysis electrodes	F1, Fz, F2, FC1, FCz, FC2, C1, Cz, C2
Sampling Rate	512 Hz or 2048 Hz

**Figure 1:**
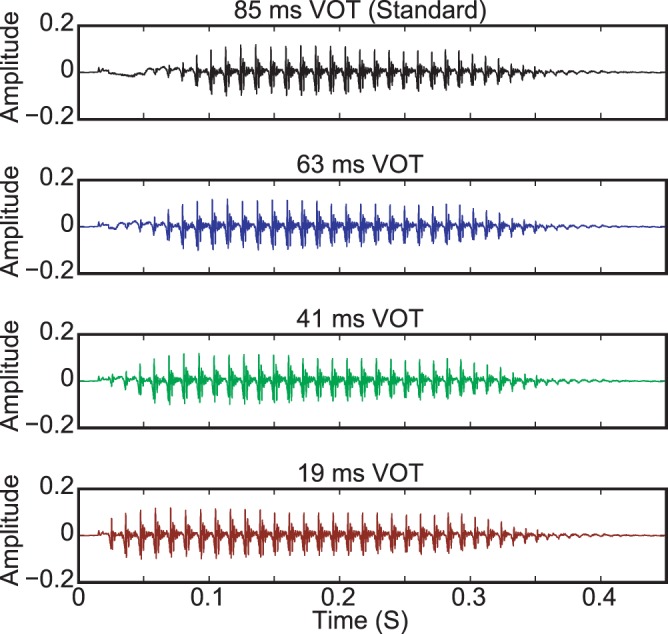
Experimental stimuli waveforms. A recording of the English CV syllable/pa/with a voice onset time of 85 ms was used as the standard stimulus during EEG recordings. The three deviant stimuli were created by removing successive 22 ms portions of the aspirated period prior to voice onset in the original 85 ms VOT standard stimulus, and by inserting additional periods of voicing to preserve the duration of each stimulus. The onset of the initial plosive burst was preserved for all of the stimuli.

Four consonant-vowel (CV) syllables representing an English language stop consonant continuum were used as stimuli during the EEG measurements. A recording of the CV syllable/pa/spoken by a male native-English speaker with a Voice Onset Time (VOT) of 85 ms was used to create the other three stimuli by removing successive 22 ms portions of the aspirated portion of the original recording prior to voice onset. Thus, the VOTs of these stimuli were 63 ms, 41 ms and 19 ms. The duration of these stimuli were preserved by inserting additional periods of voicing in the voiced portion of the recording. Waveforms of the four stimuli are presented in Fig. 1.

The purpose of this manipulation was to produce a continuum which sounded progressively more like/ba/to native speakers of English. In the English language, voiced and voiceless stop consonants (e.g./d/vs./t/,/b/vs./p/) are primarily distinguished from one another on the basis of VOT, while in the Dutch language the voiced and voiceless stop consonants are primarily distinguished by the presence of pre-voicing [37], [38]. Results of the original study indicated that both groups perceived the 63 ms VOT stimulus as/pa/(within-category relative to the 85 ms VOT stimulus) and the 19 ms VOT stimulus as/ba/(across-category). A between-groups difference was observed with respect to the 41 ms VOT stimulus, which was more likely to be perceived as/ba/by native-English speakers and as/pa/by native-Dutch speakers [36]. In other words, the 41 ms VOT was located near each of the two groups’ category boundaries, but fell on opposite sides.

During EEG measurements, these stimuli were presented in pseudorandom oddball sequences containing a standard stimulus (always the 85 ms VOT stimulus) and one of three deviant stimuli (see Table 1 for details of the oddball sequence parameters). There were three different EEG measurement conditions, which will be referred to subsequently using the name of the deviant stimulus which was used: ‘63 ms VOT deviant’, ‘41 ms VOT deviant’ and ‘19 ms VOT deviant’. During the EEG measurements, auditory stimuli were presented over loudspeakers while participants watched self-selected silent movies. Participants were instructed to ignore the auditory stimuli and to attend to the movie. This type of passive listening paradigm is typically used in conjunction with oddball stimulus sequences to elicit the MMN component of the auditory ERP [7], [8]. After the passive oddball procedure, all participants completed a two-alternative forced-choice task (without EEG measurement) in which they actively identified the stimuli on the/ba-pa/continuum.

### EEG Data Collection and Processing

Details of the EEG measurement system can be found in Table 1. Measurements were conducted inside a shielded electric cabin using a BioSemi ActiveTwo amplifier with 64 Ag/AgCl electrodes placed according to the international 10–20 system. Stimuli were presented to participants at approximately 70 dB SPL using a Monocor MKS-28 stereo loudspeaker system. Raw EEG data was measured along with left and right mastoid leads, horizontal and vertical EOG at a sample rate of either 512 or 2048 Hz. Filtering, referencing and additional preprocessing was performed offline, as described below.

For the present analysis, EEG data measured in each of the three deviant stimuli conditions were processed in non-overlapping epochs ranging from −200 ms before stimulus onset to 1000 ms post stimulus onset. Only epochs collected during trials containing a deviant stimulus and the standard trials immediately preceding them were selected for analysis, meaning an equal number of standard and deviant trials were analyzed in each condition. In each epoch, a spherical-spline interpolation procedure [39] was used to repair individual EEG channels whose power in the 50Hz band exceeded 1000 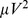 or whose offset exceeded ±25 mV. An average of 2.86 channels were repaired per epoch (St. Dev. = 1.95). The data were then resampled to 128 Hz, and an independent component analysis (using the infomax ICA algorithm as implemented in the ‘runica’ function of the EEGLab toolkit [40]) was performed on each participant’s data in order to identify and remove components containing non-EEG artifacts such as muscle or eye movements [41]. Only components which accounted for more than 1% of the overall variance in the data were considered for removal. For each of the components under consideration, the variance in each epoch of data was calculated. The mean variance across epochs was then calculated for each component. Components whose mean variance exceeded a threshold set to the average variance across all considered components were then visually inspected to verify that their time course and topography were typical of non-EEG artifacts such as neck and eye movements (highly focal spatial distribution, large amplitude). Incremental adjustments to the threshold were then made on a per participant basis to ensure that components including non-artifactual activity were not removed. This approach is similar to that used in a previous analysis of individual auditory ERPs by Bishop and Hardiman [42]. An average of 5.14 components (St. Dev. = 2.01) were removed from each participant’s data. Following the removal of these components, data were reprojected onto the measurement channels, and any epochs containing activity exceeding ±75 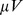 relative to the mean activity in the 100 ms window preceding stimulus onset were also removed from the dataset. On average, 97% of the analyzed epochs (St. Dev. = 3.7%) and at least 70 trials per stimulus in each of the three conditions remained following artifact rejection for all participants. Finally, data were band-pass filtered between 1 and 25 Hz, re-referenced to the average of the two mastoid leads, and baseline-corrected using the mean amplitude of the data in the 100 ms window preceding stimulus onset. All preprocessing was done using the Fieldtrip toolbox [43] in MATLAB. All subsequent classification analyses made use of EEG data in the time range between 0 and 700 ms relative to stimulus onset.

### Classification Analyses

Data collected for both native-language groups in each of the three measurement conditions were analyzed using receiver operating characteristics (ROC) analysis. Typically used for problems in the domain of signal detection theory, ROC analyses are often used to analyze both the performance of classifiers [44] as well as the discriminability of feature distributions [29], [45]. Here, we use area-under-the-ROC-curve (AUC) scores to quantify the separability of one-dimensional spatio-temporal feature distributions. These scores fall in the range of [0,1], with a score of .5 representing the no-discrimination line in the ROC graph.

Individual participant’s single-trial EEG data (64 channels×90 samples per epoch) were used to train a set of quadratically regularized linear logistic regression classifiers [46]. The regularization term is needed to limit the complexity of the classifier which prevents over-fitting in the high-dimensional input feature space [47]. To find the optimal regularization strength (or equivalently classifier complexity), a simple grid search with strengths of [.001.01.1 1 10 100] times the total data variance was used, as empirically this range has been found to give high performance.

A series of within-participants analyses were carried out to determine whether differences in the perceived categories of pairs of phonemes influenced single-trial decoding performance. To this end, a separate analysis was performed using data collected in each of the three stimulus conditions: ‘63 ms VOT deviant’, ‘41 ms VOT deviant’ and ‘19 ms VOT deviant’. These names will be used subsequently to refer to each of the within participant analyses. All of the within-participant analyses investigated a binary comparison of single-trial EEG data collected during standard trials (always the 85 ms VOT stimulus) and deviant trials in a given measurement condition. A fourth analysis was performed which included all of the data collected across conditions for each individual participant. The results of this analysis were used to compare mean decoding performance for each of the four stimuli with the individual behavioral identification scores collected in [36] using the same stimuli.

In each analysis, an equal number of epochs of data recorded during the presentation of a deviant stimulus and the standard stimulus immediately preceding it represented the two classes in a binary classification problem. On average, 202.5 consecutively recorded trials (St. Dev. = 42.2) were available for each of these classification analyses. All of the within-participant analyses utilized a ten-fold cross validation procedure, in which subsets of the available data were used for training and testing (90% and 10%, respectively) the classifier in each of the folds.

A subsequent analysis of the classifier decisions obtained at the single-trial level was performed in order to determine the performance benefits of using multiple trials. For this, we made use of the classifier decisions obtained for all available data epochs in the test folds of the within-participant analyses conducted for the 19 ms VOT deviant condition. Each decision represents a continuous probability 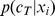 that a given data epoch 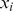 belongs to the target class 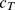. In the context of a logistic regression classifier:
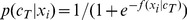
(1)


Where, 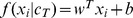 is the classifier decision value given a set of classifier weights 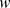 and a bias term 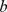. For our analysis, we combined these probabilities for non-overlapping groups of 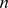 consecutive data epochs 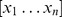 belonging to each of the two classes using a naive-Bayes formulation under the assumption of independence in the following manner:
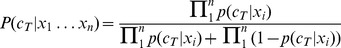
(2)


Noting that for Logistic regression 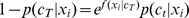, the denominator becomes:
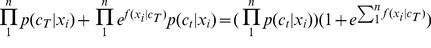
(3)and (2) becomes:



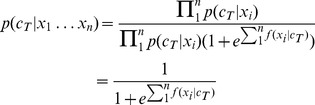
(4)Thus, one can combine decisions by simply adding together classifier decision values, which is not only simpler but also less prone to numeric round-off errors.

Another aim of the present study was to investigate whether the decoding of categorical speech perception is possible across different individuals, both within and between native-language groups. Two additional classifiers were trained on cross-participant datasets collected in the 19 ms VOT deviant condition (70 consecutive trials per class for each participant). This stimulus contrast was chosen because it represented a clear categorical distinction for both native-English and native-Dutch listeners. The first classifier was trained using data from 10 of the 11 native speakers whose within-participant classification results were significantly above chance level (see below for details), and will be referred to with the name ‘Cross-PP Native’. The second made use of all 22 participants’ data, and will be referred to with the name ‘Cross-PP All’.

Both cross-participant classifiers were trained using a double-nested cross-validation procedure in order to account for the additional inter-subject variability introduced by these datasets. Such a procedure provides a means for selecting an optimal hyperparameter for a given classification problem whilst estimating generalization performance. In each main fold of the data, one participant’s data served as a test set (for estimating cross-participant performance generalization), while the remaining participants’ data formed the classifier training set. An additional set of nested folds repeated this procedure in order to estimate the regularization parameter, with the participant whose data was used for the test set being excluded from the nested analyses.

A final series of classification analyses were conducted that aimed to decode the native language (English or Dutch) of a given participant using either EEG or behavioral data. In the previous analyses, the labels assigned to the data used for training and testing the classifiers indicated whether an individual epoch was collected on a standard or deviant trial. Here, the labels indicated whether the data belonged to a native-English or native-Dutch speaker. The classifier performance levels obtained in such an analysis indicate the extent to which the response patterns (either EEG or behavioral) obtained from the two native-language groups generalize within-group, and how well these response patterns can be distinguished from one another at the group level.

Four separate analyses were performed with each of the following data sets: concatenated single-trial data from all three measurement conditions (70 total data segments per participant), concatenated grand average data from all three measurement conditions, concatenated grand average data measured from the 63 ms and 41 ms VOT deviant stimuli (the two stimuli for which a significant between groups difference in ERP responses was observed in [36]), and the vector of behavioral identification scores for all 7 stimuli measured in the categorization task in [36]. An additional analysis combined the single-trial predictions across trials on a per-participant basis in the same manner as previously described in equation 1. A naming scheme and description of the feature vectors used in these analyses can be found in Table 2. In each analysis, data from two participants (one from each native-language group) were used for the test set in each fold while the remaining participants’ data were used for training. This led to an eleven-fold cross-validation procedure for each of the analyses.

**Table 2:** Features used in decoding analyses of native language groups.

Analysis	Feature Vector	Description
Single-Trial	64×540	Concatenated single-trial ERPs for standard and deviant
		trials in all three measurement conditions
Single-Trial (Combined)	64×540	Combined single-trial predictions (70) per participant
Grand-Average A	64×540	Concatenated individual grand-average ERPs for standard
		and deviant trials in all three measurement conditions
Grand-Average B	64×180	Concatenated individual grand-average ERPs for 63 ms
		VOT deviant and 41 ms VOT deviant
Behavioral	1×7	Mean individual behavioral responses to the
		stimulus continuum used in the original study

Feature vectors are described in terms of [channels]×[time points], with the exception of the behavioral analysis, which included mean individual responses to each of the 7 stimuli in the continuum used in the original study by Brandmeyer et al.

### Statistical Analyses

The significance levels of individual participant’s classification results in both the within- and cross-participants analyses were determined based on the estimated binomial confidence intervals for the number of data epochs available [48]. The same procedure was used to evaluate the results of the native-language decoding analysis. Two-way repeated-measures ANOVAs with either stimulus condition (factor levels: 63 ms VOT, 41 ms VOT and 19 ms VOT) or data set (factor levels: ‘individual’, ‘Cross-PP Native’ and ‘Cross-PP All’) as within-subjects factor and native language (factor levels: ‘English’ and ‘Dutch’) as a between-subjects factor were used to determine whether these variables influenced classifier performance. Subsequent within- and between-subjects comparisons were carried out using paired-samples and independent-samples t-tests, respectively.

## Results

**Figure 2:**
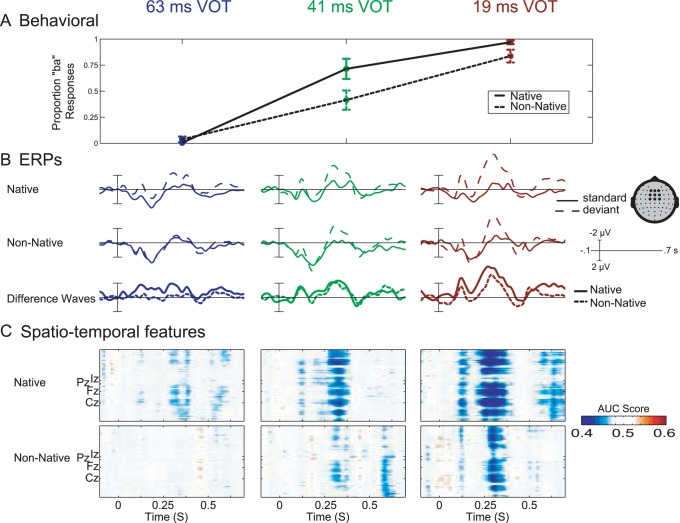
Group level behavioral and ERP responses. a) Mean behavioral identification scores for native and non-native speakers for the three deviant stimuli. b) Group-level ERPs for both the standard and deviant stimuli are presented in each of the three measurement conditions for both native-English and native-Dutch participants. Responses are averaged across nine fronto-central electrode locations, indicated by the large dots in the scalp map presented above (see also Table 1). In addition, difference waves have been derived for each language group by subtracting the grand-average responses to the standard stimulus from that of the deviant stimulus in each of the measurement conditions. c) Area under the ROC-curve scores for spatio-temporal features across the three deviant conditions for both native and non-native participants. The relative locations of four midline electrodes are indicated for reference.

A summary of the behavioral results from [36] for the stimulus conditions analyzed in the present study are presented in Fig. 2a. Grand averaged ERP responses to the standard and deviant stimuli across the three measurement conditions for both the native-English and native-Dutch groups are presented in Figure 2b, along with difference waves obtained by subtracting the grand average ERP for the standard stimulus from that of the deviant stimulus in each condition. AUC scores for spatio-temporal features in the analyzed data are presented for native-English and native-Dutch speakers in each of the three measurement conditions in Fig. 2c. ERPs collected for deviant stimuli were primarily characterized by enhancements of three negative components relative to the ERPs collected for the standard stimuli immediately preceding them: the N1, the N2 (the time interval where MMN analysis was performed in [36]) and a late negativity corresponding to the RON [19], [20]. The relative difference in amplitude of these three components is most easily seen in the difference wave plots in Fig. 2b. These same time points are also visible the AUC scores plotted in Fig. 2c. Generally speaking, the differences in the response amplitudes of these components in the standard and deviant ERPs increased as a function of the distance in VOT between the standard and deviant stimuli, with differences being the largest in the most deviant (19 ms VOT) condition.

### Within-participant Classification of Phoneme Contrasts

**Figure 3:**
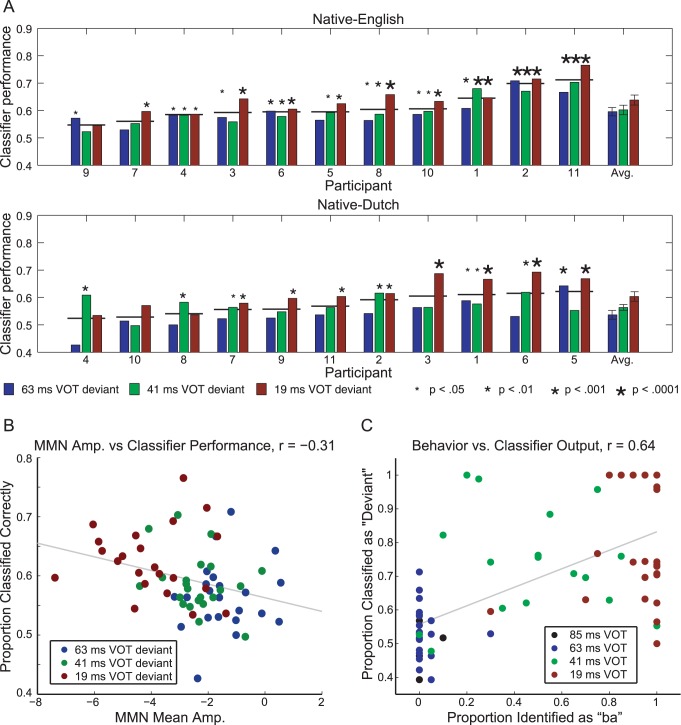
Within-participant classification analyses. a) Classification rates for native and non-native participants for each of the three stimulus conditions along with group averages (shown with error bars). Participants are sorted based on the averaged results of the three analyses, as indicated by the horizontal lines. Asterisk size indicates the significance level of the result in each of the three conditions. b) Scatter plot of classifier performance with respect to the mean amplitude of the MMN component of individual ERPs measured in the study by Brandmeyer, Desain and McQueen [16]. c) Scatter plot of mean classifier decision rates per condition with respect to behavioral decisions in the identification task reported in that study.

The results of the within-participant analyses, along with group means and significance levels for individual results, are presented in Figure 3a. A significant main effect of stimulus condition (63, 41 or 19 ms VOT deviant) was found (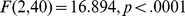), along with a marginal effect of native language group (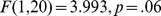). On average, classification rates increased as the difference in VOT between the standard/pa/and the deviant stimulus grew larger, with classification rates for the 19 ms VOT deviant (across-category) significantly higher than those of both the 63 ms VOT deviant (within-category) (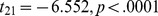) and the 41 ms VOT deviant (category-boundary) (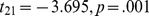). Additionally, mean single-trial classification rates in each of the three analyses were higher overall for the native-English speakers than for the native-Dutch speakers, with the difference reaching significance for the 63 ms VOT deviant (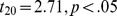).

Figure 3b plots the relationship between individual classifier performance across different conditions and the mean individual MMN amplitudes measured in [36] at fronto-central locations (see Table 1) in the same conditions (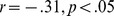), with more negative mean amplitudes tending to correspond with higher classification rates. Figure 3c plots the relationship between individual mean classifier decision rates obtained per stimulus when training a classifier using data from all three conditions (standard/pa/and the three deviant stimuli) and the individual behavioral identification scores from [36] for the same stimuli. A strong relationship between the classifier decision rates and the individual identification rates was found (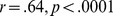), with stimuli classified as deviants more likely to be identified as/ba/by participants.

**Figure 4:**
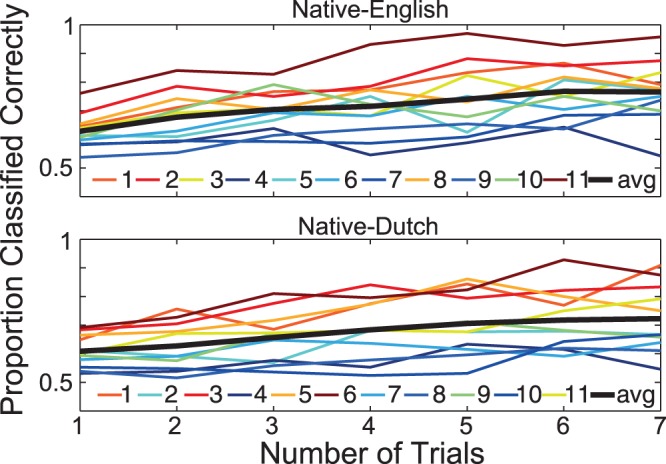
Classification across multiple trials for the 19 ms VOT condition. Multi-trial performance for individual participants in both the native-English and native-Dutch participant groups is shown using colored lines (sorted according to mean individual performance), while the average for each group is shown using a thick black line. On average, performance increased when including additional trials. Participants with relatively high single-trial classification rates tended to show additional improvement when decisions were based on additional trials, while participants with low single-trial classification rates showed less benefit from the inclusion of additional trials.

An additional analysis of the classifier predictions obtained in the 19 ms VOT condition was performed to determine the performance benefits obtained when combining classifier predictions from multiple successive data epochs. These results are plotted in Figure 4. As one would expect, classification rates increased on average with each additional trial of data that was included. Moreover, the benefit gained from an increased number of trials was related to the single-trial classification rate. Participants with high single-trial classification rates reached rates above 0.9 when using 7 trials of data, while participants with low single-trial rates showed relatively little improvement and even a drop in performance.

### Cross-participant Classification of Phoneme Contrasts

**Figure 5:**
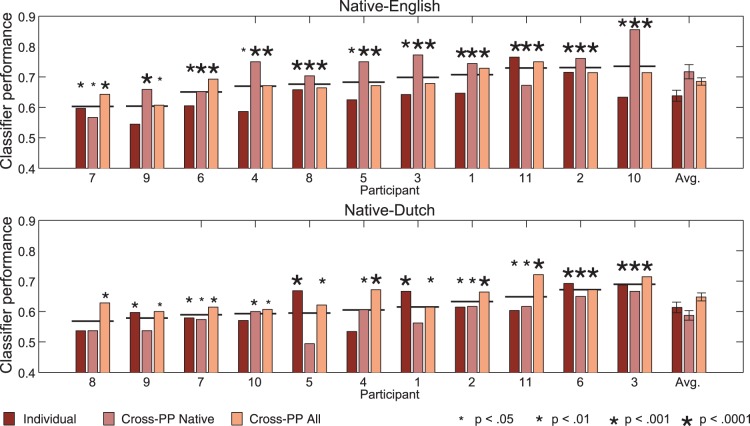
Cross-participant classification analyses. Classification rates for native and non-native participants for the two classifiers trained on cross-participant data sets using the 19 ms VOT deviant, along with individual rates from the within-participant classification analysis of the same deviant condition. Results for each of the three datasets are indicated using different colored bars. Participants are sorted based on the averaged results of the three analyses, as indicated by the horizontal lines. Group averages are also shown with error bars. Asterisk size indicates the significance level of a given ndividual result.

Cross-participant classification results are plotted in Figure 5, along with the individual within-participant results for the same condition (19 ms VOT deviant, across-category). Significant main effects of data set (‘individual’, ‘Cross-PP Native’ or ‘Cross-PP All’, 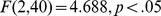) and native language (
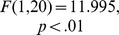
) were found along with a significant interaction of the two variables (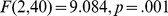). On average, classifier performance was significantly higher when trained using data from all participants than when trained using individual participant’s data sets (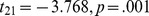). This is possibly due to the fact that a larger number of examples were used in training this cross-participant classifier. It might also be the case that the nature of the between-participant variability reflects non-essential sources of information, which in turn help prevent the classifier from over-fitting the training set data in the individual folds. No significant difference was found in classifier performance when trained on the ‘Cross-PP Native’ dataset as compared to either the within-participant classifier performance nor the classifier trained on ‘Cross-PP All’ dataset. However, when comparing the mean rates of the two groups across data sets, classifier performance was significantly higher for native-English speakers when trained using the ‘Cross-PP Native’ dataset (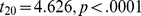), and marginally so when trained on the ‘Cross-PP All’ dataset (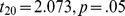).

**Figure 6:**
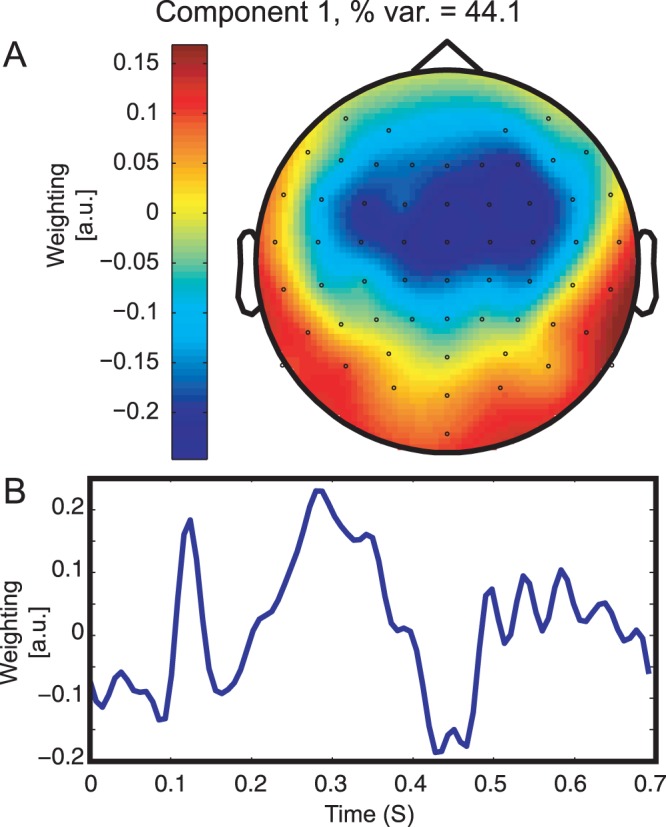
Topography and time-course of first component obtained through a singular value decomposition of the classifier weights trained on data from all 22 participants. The data are presented in an arbitrary unit scaling.

A singular value decomposition of the weight matrix of the classifier trained on the ‘Cross-PP All’ dataset was performed to identify the topography and time course of the components which explain the largest portion of the classifier’s overall performance. The largest of these components is plotted in Figure 6. As can be seen, this component explains about 44% of the variance in the classifier weighting matrix, has a negative fronto-central distribution typical of the MMN response [8], and which highly resembles the difference wave time courses during the peak of the ERP responses for the 19 ms VOT deviant condition presented in Figure 1. Moreover, a high correlation (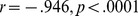) between this component’s time course and the average of the difference waves of the ERPs for all participants at the same time points indicates a strong relationship between the classifier weighting matrix and the ERPs.

### Decoding of Native Language

**Figure 7:**
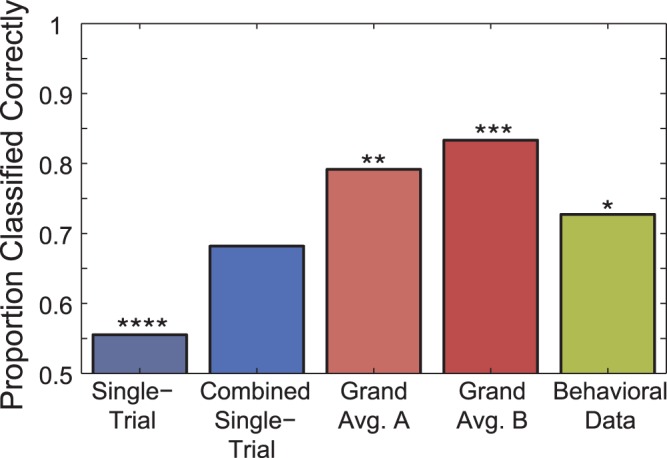
Prediction of native language on the basis of electrophysiological and behavioral data. Five decoding analyses aimed at predicting the native-language of a given-participant on the basis of their measured data were carried out. Two analyses made use of concatenated single-trial EEG data from each of the three measurement conditions. The first of these analyses determined single-trial classification rates using this data set, while the second combined the single-trial predictions (70 total trials) for each participant’s data obtained when it was used as a test-set during the classification analysis. Two additional analyses made used of concatenated individual grand-averaged ERPs. One utilized both standard and deviant stimulus ERPs collected in all of the three measurement conditions, while the other included only the deviant ERPs measured using the 63 and 41 ms VOT stimuli. A final analysis was performed using a vector of seven mean behavioral identification scores collected for each participant in the original study by Brandmeyer et al. Significance levels shown using asterisks (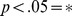, 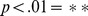, 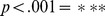, 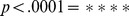), and are based on the number of observations available for each of the five data sets. For the single-trial analysis, 1540 data points (70 per participant) were available, while for the remaining four analyses, 22 data points (one per participant) were available.

The mean results for each of the five analyses are plotted in Figure 7. Classifier performance was significantly above chance for four of the five data sets which were analyzed: single-trial data, individual average ERP data, individual average ERP data for 63 and 41 ms VOT deviants, and individual behavioral data. The exception was when using the combined single-trial predictions. The highest overall rate of 83% was attained when using only averaged individual ERP data from the 63 ms and 41 ms VOT deviant stimuli. These were the two conditions which showed a significant between-groups difference in the ERP analysis from the original study [36]. Classifier performance was slightly lower when using the ERP data from all measurement conditions, followed by the analysis in which the vector of mean individual identification scores collected during the original study was used.

## Discussion

The present study investigated the outcomes of a series of multivariate pattern classification analyses of EEG data collected during passive speech perception of English phonemes by native and non-native listeners. These analyses addressed two principal research questions: 1) Is it possible to decode stimulus categories from single-trial EEG data elicited using different speech sound contrasts for native-English and native-Dutch speakers? 2) Is it possible to decode these same stimulus categories across individual participants, either within or between native-langauge groups?

### Within-participant Analyses

The results of the within-participant analyses demonstrate that single-trial EEG measurements of brain responses to phonemes contain sufficient information to decode speech sound categorization, and that the performance of such analyses improved across conditions representing increasingly salient phonetic contrasts. As such, the results confirm that the within-participants trends previously observed in analyses of grand-averaged ERP data are also present at the single-trial level [36]. In the case of the 19 ms VOT condition, which employed a stimulus contrast that clearly represented two distinct phonetic categories for both native-English and native-Dutch participants, classifier performance was significantly higher than conditions which employed a within-category (63 ms VOT) or ambiguous (41 ms VOT) contrast. Previous research findings have shown enhancements of different components of the auditory ERP to across-category deviant stimuli as compared to within-category deviant stimuli, including the MMN [10] and the N2/P3 complex [15]. Enhancements of these components in deviant trials would in principal increase the amount of information available during pattern classification, leading to higher overall performance.

A marginally significant effect of native language was also observed, suggesting an overall difference in decoding performance across the two groups. In general, decoding rates were higher for the native-English speakers as compared with the native-Dutch speakers, with this difference reaching significance for the 63 ms VOT condition. When looking at the AUC scores presented in Fig. 2c, we see a clear difference in the amount of discriminative information available between the two language groups, not only in the 63 ms VOT condition, but across all three conditions. This could in part explain the overall differences which were observed in decoding performance between the two groups. It is worth noting again that the native-Dutch group of participants were in fact highly proficient English speakers, having undergone 6 years of coursework as part of their high school curriculum. The fact that differences in decoding performance are still observed highlights the formative role played by language learning in early childhood in shaping our long-term perception of speech [49]–[54].

A modest correlation was observed when investigating the relationship between individual decoding results and individual mean MMN amplitudes measured in the original analysis in [36]. The fact that this relationship was not stronger may be because the multivariate analysis also included time points and scalp locations which were not part of the original analysis in [36]. While the individual MMN amplitudes reflect the activity in a 50 ms time window around the peak of the MMN difference wave at fronto-central locations between 200–400 ms following stimulus presentation, the classification analysis included data from all 64 recording channels at time points between 0 and 700 ms post-stimulus onset. As such, it included additional ERP components including the N1, P3a and RON. Previously work on single-trial classification of ERP components has shown that the inclusion of components at different time intervals within an ERP provides additional information when distinguishing different classes of signals, leading to an improvement in classifier performance [55]. So while a significant relationship was observed between the MMN component of individual ERPs and the results of the within-participant classification analyses, it would appear that decoding performance is also influenced by a broader hierarchy of cognitive processes underlying the responses observed at different time points.

A much stronger correlation was found when examining the relationship between the individual behavioral data collected in [36] and the per stimulus classification performance observed when training a classifier using data from all three conditions. This seems to suggest an overlap in the functional organization underlying both the perceptual decision making process during behavioral identification and the single-trial brain responses used by the classifier during its decision-making process. This result is perhaps most interesting when we consider the fact the behavioral identification measurements reflect an active process (responding to individual stimuli) while the EEG measurements reflect a passive process (perception of sound sequences while viewing a film).

Within-participant single-trial classification rates were comparable with the average rates reported in [35]. The results of that study also showed a graded pattern of results depending on which manipulation (auditory space, syntax or both) was present in the experimental stimulus. Here, the graded responses are observed relative to a continuous change in one specific acoustic feature of the deviant stimuli (VOT), as well as with respect to the native language of individual participants. When compared to the average single-trial rates observed in experiments making use of an active auditory listening task [28], [29], [31], [34], the rates reported here are substantially lower. This is most likely due to the fact that the tasks in the studies just mentioned were designed to elicit the P300 response, which has a substantially higher amplitude (10–20 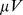) than the ERP components elicited during passive listening, such as the MMN (0.5–5 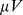) [8], [56]. Such increases in signal amplitude lead to a higher signal-to-noise ratio, and improve classification performance.

It was also shown that this performance could be improved through the inclusion of additional trials. Performance increased on average with each additional trial that was included, reaching above 95% correct for some participants when 7 trials were included in the classifier’s decision. However, the relative benefit in classification performance which was achieved through the use of additional trials was also a function of individual participant’s single-trial classification rates. While individuals with relatively good single-trial classification rates tended to show the most improvement across trials, participants with low single-trial classification rates did not show much benefit when including additional trials, with performance sometimes being even lower than the single-trial rates. This would seem to point to a general lack of discriminative information in the single-trial EEG data for some participants. Previous multivariate pattern classification analyses of EEG-data collected in an auditory paradigm and using multiple-trials have also shown similar results [33]. Such differences may be due in part to what has been referred to as ‘BCI illiteracy’, in which some participants do not show a neural signature of interest for a given task [57]. Previous studies on individual MMN responses have also demonstrated that not everyone will show a clear MMN component despite exhibiting normal auditory perceptual abilities [42].

### Cross-participant Analyses

One of the goals of the present analysis was to determine the amount of individual overlap in the functional brain organization underlying the perception of the phonemes used during EEG measurements, both within and across native-language groups. When using a classifier trained on data from 10 of the 11 native-English participants collected in the 19 ms VOT condition (‘Cross-PP Native’), a difference in the classifier’s performance was observed for the two language groups. While performance improved for native-English speakers relative to the within-participant analysis (64% vs 72% correct), performance decreased for native-Dutch speakers (61% vs 59%). In contrast, when using a classifier trained using data collected in the same condition from all 22 participants (‘Cross-PP All’), a significant overall improvement was observed for all participants relative to the within-participant analysis. Here the performance benefit for native-English speakers was slightly less as compared with the benefit seen when using a classifier trained using only data from native-English speakers. This seems to indicate a discrepancy in the extent to which features present in the single-trial data of native-English speakers are utilized by the two cross-participant classifiers, and that features present in the single-trial data of the native-Dutch speakers do not completely overlap with those of the native-English speakers.

Previous work using fMRI to investigate differences in the functional neuroanatomy of language processing between native and non-native speakers suggests that, while both groups rely on the same cortical network, non-native speakers show enhanced activation in some regions relative to native speakers [58]. Studies using ERP measurements have suggested an enhancement of ERP components related to the processing of both acoustic features [36] and categorical information [4], [5] measured with native speakers relative to non-native speakers. Combined, these results suggest both similarities in the functional organization of language processing in native and non-native speakers as well as differences in the distributed activation patterns for specific linguistic tasks. The present cross-participant analyses provide additional support for this view. They are also in line with previous cross-participant classification analyses presented in [33], [35], which showed either equivalent or improved classification performance when using cross-participant data sets as compared to within-participant datasets. As was the case with the results presented in [33], the overall improvement in performance here may be due to the increased amount of training data available in the cross-participant analysis.

This study also presented the results of a set of cross-participant classification analyses that focused on the native language of participants. Analyses that made use of single-trial ERP data were less successful at determining the native language of a given participant than those which made use of individual behavioral data. However, analyses which made use of individual grand-averaged ERPs showed better native-language classification than the analysis using behavioral data, with the best overall performance obtained when using ERPs measured in response to the 63 ms VOT and 41 ms VOT deviant stimuli. These were the two conditions which showed a significant between-groups difference in MMN response amplitude in the original study [36]. These results suggest that our brain responses to speech may reveal more about our linguistic background than our behavioral responses to it. They also align nicely with the results of the cross-participant analyses discussed above, in that they also suggest differences in the distribution of activation patterns measured in response to speech stimuli between native-English and native-Dutch speakers.

### BCI Paradigms Based on Speech Perception

The use of multivariate pattern classification methods to identify differences in the characteristic brain responses generated by individual members of groups with differing perceptual profiles could have potential applications in both education and clinical settings. A new class of BCIs has recently been described, called passive BCIs, which combine cognitive monitoring with the real-time decoding methods typical of BCIs [59]. A passive BCI based on the listening paradigm used in this study could be used to monitor the brain activity underlying auditory perception. In educational settings, such a system could be used to ascertain whether one’s brain responses to foreign speech sound contrasts resemble those of a native speaker or not. Likewise, in clinical settings, characteristic abnormalities in the MMN component have been reported for a wide-variety of clinical populations, including children with specific language impairment and individuals diagnosed with schizophrenia [7]. In turn, the use of an appropriate BCI may be able to reduce the measurement times which are needed in order to ascertain whether an individual’s brain responses fit a particular neurological profile. However, some caution is needed when considering such approaches. Many ethical issues arise when considering the applications made possible by single-trial decoding approaches, including the unwilling extraction of personal information from measurements of brain-activity and their potential (mis)use in criminal investigations [3], [23], [60].

The present results also suggest that BCIs which directly support language learning through neurofeedback have potential. Neurofeedback provides real-time information about brain activity as measured using EEG or fMRI, providing users with a mechanism to modulate activity related to specific brain structures or cognitive states [61]–[65]. In a recent study [66], multivariate methods were employed in conjunction with fMRI measurements of activity in striate and extrastriate cortical regions during visual perception of simple orientation stimuli, and were subsequently used to provide participants with a neurofeedback signal based on decoded brain activity from these same regions. Following 5–10 days of neurofeedback training, participants showed enhanced visual perception of stimuli corresponding to the trained activation patterns.

This type of induced perceptual learning may also be possible using decoded-EEG neurofeedback based on the evoked responses underlying speech perception. Such a system would, in principle, provide users with real-time information regarding their brain’s ongoing responses to unfamiliar foreign speech sound contrasts, as reflected in the MMN and other components of the auditory evoked response. Research on the time course of language learning and associated changes in brain responses has shown that the MMN response develops prior to changes in behavioral responses associated with the successful discrimination of foreign phoneme contrasts [67]. Thus it would be possible to provide users with neurofeedback in a time span where the perceptual learning process is still ongoing. The results of the multi-trial analysis presented above also suggest that the reliability of such feedback could be regulated by combining classifier decisions across a sufficient number of subsequent trials. Moreover, it may also be possible to make use of classifiers trained on cross-participant data sets from, for instance, native speakers, for use in neurofeedback paradigms intended for second-language learners. While additional research would obviously be needed to verify the merit of this approach, the results presented here in conjunction with those from [66] suggest that such an approach is possible. Many challenges remain in the development of such a system. For example, it is still an empirical question how high the single-trial classification rate has to be to support language learning. Nevertheless, the above-chance single-trial classification reported here is promising. It indicates that, at least with respect to the multivariate pattern classification that would be required, a neurofeedback system for the training of speech perception is feasible.

## Conclusion

The present study has shown that both within- and cross-participant decoding of evoked responses measured during speech perception is possible, with the results being a function of both the relative size of the contrasts employed as well as the phonological status of the contrast for a given listener. Moreover, the results indicate that, while the functional brain organization underlying speech perception may involve the same fundamental networks in native and non-native speakers, differences in the relative distribution of activation patterns influence the outcomes of the multivariate analyses for native and non-native speakers. On the basis of these results, we suggest that these methods can be used for developing novel BCI applications related to second language learning.
